# Trend and Burden of Suboptimal Breastfeeding in Children Under Five Years of Age in 1990–2021: A Systematic Analysis for the Global Burden of Disease Study 2021

**DOI:** 10.3390/nu17071134

**Published:** 2025-03-25

**Authors:** Bengui Jiang, Kelly Lin, Nicholas Buys, Bei Zhang, Yanfei Qi, Jing Sun

**Affiliations:** 1 Ningbo Women and Children’s Hospital of Ningbo University, Ningbo 315010, China; jiangbengui@126.com; 2 Ningbo Clinical Research Center for Gynaecological Diseases, Ningbo 315010, China; 3 Rural Health Research Institute, Charles Sturt University, Orange, NSW 2800, Australia; kelly.lin@griffithuni.edu.au (K.L.); sanx1m@163.com (B.Z.); 4 School of Medicine and Dentistry, Griffith University, Southport, QLD 4215, Australia; 5 School of Health Science and Social Work, Griffith University, Gold Coast Campus, Southport, QLD 4215, Australia; n.buys@griffith.edu.au; 6 Centenary Institute, The University of Sydney, Sydney, NSW 2050, Australia; j.qi@centenary.org.au; 7 Data Science Institute, University of Technology Sydney, Sydney, NSW 2000, Australia

**Keywords:** suboptimal breastfeeding, chronic malnutrition, breastfeeding

## Abstract

**Background/Objectives**: Breastfeeding is a cost-effective early child health intervention that has been identified as a protective factor against adverse child health outcomes. However, as estimated by previous epidemiological studies, the prevalence of breastfeeding in most countries around the world is below the recommended levels established by the World Health Organization. This study aims to assess the changes in suboptimal breastfeeding mortality, disability-adjusted life years (DALYs), and years lost to disabilities (YLDs) on a global, regional, and national level from 1990 to 2021. **Methods**: Data regarding suboptimal breastfeeding in children under 5 years of age from 1990 to 2021 were extracted from the Global Health Data Exchange query tool. Data from 204 countries and territories countries were classified into 5 regions based on the sociodemographic index (SDI) and 21 Global Burden of Disease (GBD) regions according to geographical contiguity. The average annual percentage change (AAPC) was calculated to assess changes in the trends of suboptimal breastfeeding DALYs, YLDs, and mortality in the past 30 years. **Results**: Countries with high–middle (AAPC = −0.94, 95% CI = −0.95 to −0.93) SDI scores had the greatest degree of improvement in both suboptimal breastfeeding mortality from 28,043.47 to 1128.24 and disease burden from 43,202.94 to 4428.47, while countries with high (AAPC = −0.83, 95% CI = −0.86 to −0.81) and low SDI (AAPC = −0.63, 95% CI = −0.71 to −0.55) scores showed the least improvement from 16,775.75 to 5930.56 and 49,522.23 to 32,881.08, respectively. **Conclusions**: Significant global improvements in suboptimal breastfeeding mortality and morbidity have occurred in the last 30 years. However, the extent of improvement differs significantly across nations, while some countries also showed no improvements or increased suboptimal breastfeeding mortality and disease burden. Nation-specific policies that account for cultural practices and economic conditions are required to target vulnerable mothers that are unable to achieve optimal breastfeeding.

## 1. Introduction

Breastfeeding is a cost-effective early child health intervention that has been identified as a protective factor against adverse child health outcomes including diarrhea, pneumonia, poor cognitive development, and premature mortality [1,2]. The health benefits of breastfeeding extend to both mothers and babies in high-income and low-income settings [1,2]. However, as estimated by previous epidemiological studies, the prevalence of breastfeeding in most countries around the world is below the recommended levels established by the World Health Organization (WHO) [2]. Suboptimal breastfeeding has been further identified as the second largest risk factor for children under five, accounting for 47.5 million disability-adjusted life years (DALYs) lost in 2010 and 11.6% of under 5 mortalities [3]. Given the importance of exclusive breastfeeding in early childhood, one of the six WHO global nutritional target is to increase the rate of exclusive breastfeeding under 6 months to at least 50% by 2025 [4].

The current recommendations of breastfeeding from the WHO have suggested that, when possible, exclusive breastfeeding is recommended for the first 6 months of life, and continued breastfeeding for at least the first 2 years of life, with complementary foods being introduced at 6 months postpartum [5]. In this study, ‘suboptimal breastfeeding’ is defined as a combination of non-exclusive breastfeeding, where infants receive other foods or liquids in addition to breastmilk before six months of age, and discontinued breastfeeding, which occurs when breastfeeding ceases before the recommended two years, based on recommendations from the WHO [5].

Previous studies have estimated that by achieving optimal levels of breastfeeding prevalence, 13% of child mortalities in low-income countries can be prevented [6]. Optimal nutrition in the first 1000 days of life is critical to ensure that nutritional needs are met to allow the child to develop, learn, and thrive [7]. Thus, in low-to-middle-income countries (LMICs), improving breastfeeding has become a priority as breastfeeding is a cost-effective intervention that can easily reach populations with limited access to advanced health infrastructures. Barriers to breastfeeding is multifactorial and can be assessed using the Socio-Ecological Model [8]. Individual factors from the mother and babies interact with environmental systems to influence breastfeeding [8]. Using the Socio-Ecological Model, global authorities have developed codes and resolutions to support mothers to improve the prevalence of breastfeeding [9,10].

Two main groups of codes have been developed: firstly, focusing on maternity protection through paid maternity leaves and support for continued breastfeeding practices upon returning to work through the International Labor Organization (ILO) Maternity Protection Convention, 2000; and secondly, limiting the inappropriate marketing practices of breastmilk substitutes (BMS) and feeding bottles and teats by adopting the Code of Marketing of Breast-Milk Substitutes by the World Health Assembly (WHA) [9,10]. Despite the establishment of recommendations and codes using evidence-based research, the compliance and adoption of either convention or code is heterogenous across nations and regions. Past reviews have identified that nearly 50% of the countries have not met the minimum 14 weeks of paid maternity leave [10], while only 38% of WHO member states have clear guidelines to monitor compliance for the Code of Marketing of Breastmilk Substitutes [11].

Differences in cultural practices and economic conditions between countries lead to variations in national policy enforcements on achieving optimal breastfeeding to reduce maternal and child morbidity and mortality. Analyzing trends in suboptimal breastfeeding will help raise awareness on nations that require further support to achieve optimal breastfeeding. Recent studies have focused on examining the coverage and enforcement of national policies in accordance with the established global codes [11,12]. However, to the author’s knowledge, recent changes and trends in suboptimal breastfeeding have not been examined. To understand the effectiveness of national policies, trends in suboptimal breastfeeding should be assessed in addition to the policies established. Assessing global trends in suboptimal breastfeeding is also critical to help identify nations and regions at risk. Thus, this study aims to assess the changes in suboptimal breastfeeding mortality, DALYs, and years lost to disabilities (YLDs) on a global, regional, and national level from 1990 to 2021.

## 2. Materials and Methods

### 2.1. Overview

This study follows the Guidelines for Accurate and Transparent Health Estimates Reporting (GATHER) statement and the Global Burden of Disease (GBD) protocol (Supplementary Materials). The GBD study consists of a global network of collaborators that provide, review, and analyze data collected regarding causes of disability, premature death, and injury burden over time and across age groups, sexes, and location. This study aims to promote evidence-based intervention and monitor progress towards national and international health targets. Data for this study were extracted from vital registration systems, verbal autopsies, censuses, household surveys, disease-specific registers, health service contact data, and other sources. Further detailed descriptions of the methodology for GBD estimation can be found elsewhere [13].

In line with GBD methodologies, suboptimal breastfeeding in this study is composed of “non-exclusive breastfeeding” and “discontinued breastfeeding”. Exclusive breastfeeding is defined as the proportion of children who received no other food or drink in the first 6 months after birth, except for breastmilk (ORS, drops, syrups containing vitamins, minerals, or medicines were allowed in the GBD definition). Discontinued breastfeeding is defined as the proportion of children who did not receive breastmilk as a food source from 6 to 23 months after birth. Breastfeeding data were collated based on multinational survey series, country-specific surveys and reports, and the scientific literature. Further details regarding the GBD study definitions of suboptimal breastfeeding, data extraction, and processing have been described in published GBD protocols [14].

### 2.2. Data Sources

This study utilized data from the 2021 GBD study that modeled non-fatal disease burden using DisMod-MR version 2.1. This meta-analysis tool uses a compartmental model structure with different equations that helps synthesize heterogenous epidemiologic data for non-fatal disease. In this study, suboptimal breastfeeding was defined as a combination of non-exclusive breastfeeding and discontinued breastfeeding, based on recommendations from the WHO [5].

This study measured suboptimal breastfeeding disease burden through DALYs and YLDs. Suboptimal breastfeeding-associated mortality was also determined using age-standardized mortality rates (ASMRs). Data from children under 5 years of age from 1990 to 2021 were extracted from the Global Health Data Exchange query tool. We included data from 204 countries and territories. These countries were classified into 5 groups based on the sociodemographic index (SDI) and 21 GBD regions according to geographical contiguity.

### 2.3. Sociodemographic Index (SDI)

The sociodemographic index (SDI) acts as a summary measure that represents a country’s social and economic development [15]. This summary measure includes a nation’s economy as measured by lag-distributed income (LDI) per capita, mean education for those 15 and older, and the total under 25 fertility rates of nations. This index is used in the Global Burden of Disease studies because the outcomes measured by the SDI correlate strongly with health outcomes. Based on the country’s SDI score, each country has been classified into one of the five categories—high, high–middle, middle, low–middle, and low.

### 2.4. Statistical Analysis

The average annual percentage change (AAPC) was calculated using Dis-Mod II to determine the temporal trend in suboptimal breastfeeding disease burden and mortality in children under 5 years of age. Suboptimal breastfeeding DALYs, YLDs, and mortality number and rate per 100,000 persons were used for AAPC calculations to show a temporal trend of a 30-year period. A positive AAPC indicates an increased rate or number in disease burden or mortalities, while a negative AAPC indicates decreasing or improving trends. The level of statistical significance was measured using a 95% confidence interval (CI). Further details on the statistical analysis methodology used in this paper can be found in our previously published papers [16].

### 2.5. Ethics

This study drew on global, regional, and national data collected by the GBD study. Thus, no further ethics approval was required, as the study did not involve data collection, experimentation, or investigation regarding human subjects. Nonetheless, the authors of this study are aware of ethical considerations regarding secondary data analysis. Access to datasets has been granted to all authors and collaborators in this study. Secondary data accessed from GBD are anonymous and have been de-identified. Authors have further acknowledged and followed GBD protocols, while acknowledging the use of the GBD dataset throughout this study.

### 2.6. Role of Funding Source

The study funder had no role in the study design, data collection, data analysis, data interpretation, report writing, or decision to submit this manuscript for publication.

## 3. Results

### 3.1. Global

**Figure 1:**
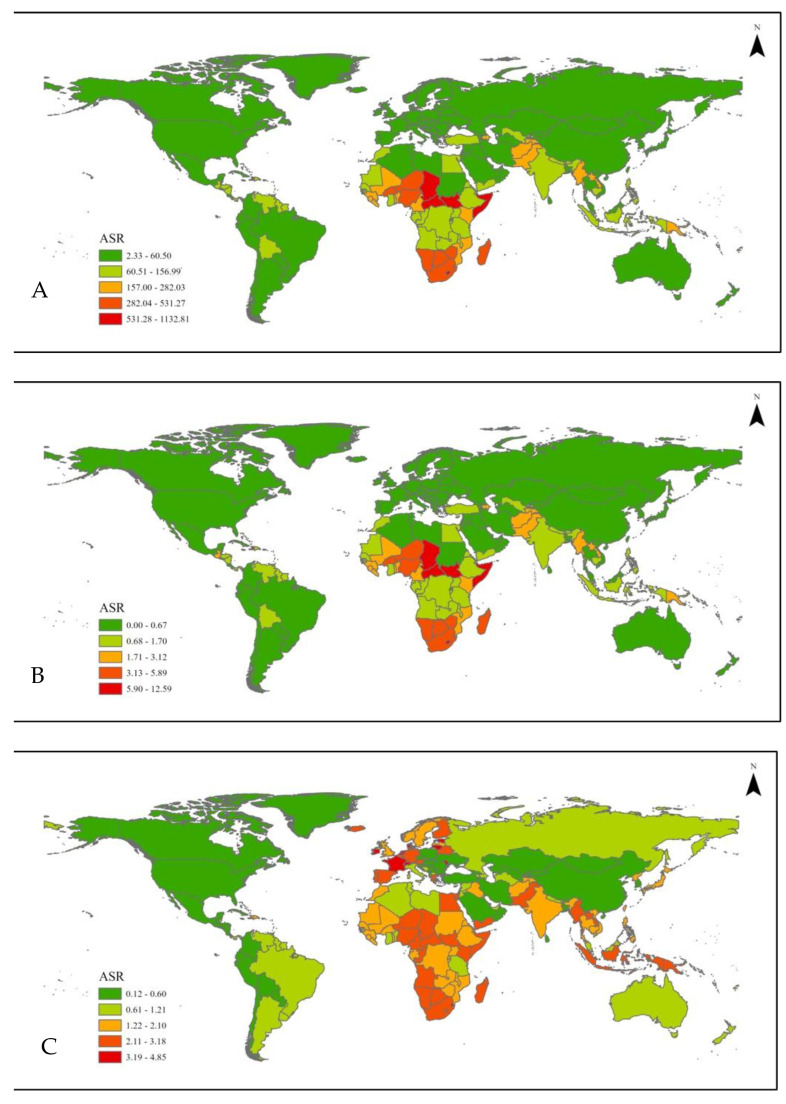
Global age standardized rate of global suboptimal breastfeeding (**A**) DALYs, (**B**) mortalities, and (**C**) YLDs.

Table 1 and Figure 1 demonstrate changes in the global burden of disease and mortality due to suboptimal breastfeeding in children under 5 years of age. A significant reduction in the suboptimal breastfeeding number of DALYs was identified with a decrease from 47,368,654.78 in 1990 to 9,161,024.86 in 2021, with an AAPC of −0.81 (95% CI = −0.84 to −0.76). The number of mortalities decreased from 532,453.25 to 101,023.15 with an AAPC of −0.81 (95% CI = −0.81 to −0.76), while the number of YLDs decreased from 283,438.12 to 96,845.1 with an AAPC of −0.58 (95% CI = −0.61 to −0.55) from 1990 to 2021.

### 3.2. Sociodemographic Index

Significant improvements in suboptimal breastfeeding mortality and disease burden were also notable in all SDI categories. Of them, countries with high–middle SDI scores had the greatest degree of improvement in both suboptimal breastfeeding mortality and disease burden, while countries with low SDI scores showed the least improvement. As presented in Table 1, the most significant reduction in the number of suboptimal breastfeeding mortalities was in children in high–middle SDI countries from 28,043.47 to 1128.24 with an AAPC of −0.94 (95% CI = −0.95 to −0.93), followed by middle SDI countries from 135,778.97 to 11,836.11 with an AAPC of −0.9 (95% CI = −0.92 to −0.88), low–middle countries from 202,867.48 to 31,831.85 with an AAPC of −0.86 (95% CI = −0.89 to −0.83), high SDI countries from 1158.91 to 265.06 with an AAPC of −0.83 (95% CI = −0.86 to −0.81), and low SDI countries from 164,270.38 to 55,868.35 with an AAPC of −0.63 (95% CI = −0.71 to −0.55).

Similar trends were also observed for morbidities associated with suboptimal breastfeeding as measured by YLDs. The most significant improvement in the number of suboptimal breastfeeding YLDs was identified in high–middle SDI countries from 43,202.94 to 4428.47 with an AAPC of −0.83 (95% CI = −0.85 to −0.82), followed by middle SDI countries from 96,955.83 to 19.015.58 with an AAPC of −0.76 (95% CI = −0.78 to −0.75), low–middle SDI countries from 76,770.74 to 34,512.35 with an AAPC of −0.52 (95% CI = −0.56 to −0.47), high SDI countries from 16,775.75 to 5930.56 with an AAPC of −0.39 (95% CI = −0.45 to −0.34), and low SDI countries from 49,522.23 to 32,881.08 with an AAPC of −0.23 (95% CI = −0.29 to −0.14). Thus, the burden of suboptimal breastfeeding remained the most significant in low SDI countries in 2021, with the least improvement observed.

### 3.3. Regional

**Figure 2:**
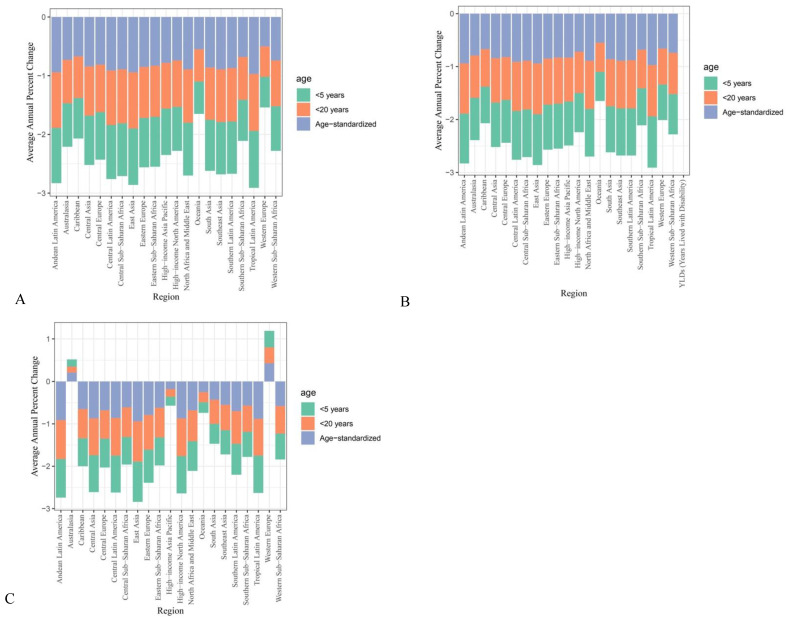
Under 5 years regional 1990 to 2021 suboptimal breastfeeding AAPCs of (**A**) DALYs, (**B**) mortalities, and (**C**) YLDs.

Regional changes in suboptimal breastfeeding trends are displayed in Figure 2. When countries were grouped based on geographical locations, East Asian regions showed the most significant improvements with a decrease in the number of suboptimal breastfeeding mortalities from 65,684.81 to 1244.34 with an AAPC of −0.97 (95% CI = −0.98 to −0.96). This was followed by the Latin American regions, including Tropical Latin America, with a decrease in the number of mortalities from 20,561.64 to 447.27 with an AAPC = −0.97 (95% CI = −0.98 to −0.96), Andean Latin America from 3755.27 to 232.92 with an AAPC of −0.93 (95% CI = −0.95 to −0.91), and Central Latin America from 14,338.26 to 1079.76 with an AAPC of −0.93 (95% CI = −0.95 to −0.91). Similar trends were identified for suboptimal breastfeeding YLDs, where East Asia exhibited the most significant improvements with a decrease from 6707.63 to 4129.27 with an AAPC of −0.97 (95% CI = −0.97 to −0.96). Substantial improvements were noted in the Latin American region, with Tropical Latin America and Central Latin America both recording an AAPC of −0.88 (95% CI = −0.98 to −0.96) with a decrease in the number of YLDs from 11,177.75 to 1141.03 and 12,322.29 to 767.78, respectively. In addition, high-income North America (AAPC = −0.88, 95% CI = −0.9 to −0.87) and Eastern Europe (AAPC = −0.87, 95% CI = −0.88 to −0.86) also showed substantial improvements in suboptimal breastfeeding YLDs.

Compared to Latin American regions, the improvements in mortality and morbidity of suboptimal breastfeeding were less significant. In the Western Sub-Saharan Africa region, the number of mortalities was reduced from 98,624.85 to 38,980.03 in 2021 with an AAPC of −0.46 (95% CI = −0.57 to −0.33), and the number of YLDs also remained high at 18,465.06 with an AAPC of −0.13 (95% CI = −0.21 to −0.02). In the Eastern Sub-Saharan Africa regions, the number of suboptimal breastfeeding mortalities decreased from 52,787.84 to 14,964.1 with an AAPC of −0.73 (95% CI = −0.80 to −0.63). Furthermore, the Oceania region was the only region that showed no significant improvements from 1990 to 2021, with a slight increase in the number of mortalities from 471.77 to 472.46 with an AAPC of −0.14 (95% CI = −0.37 to 0.18). No significant decreases in suboptimal breastfeeding-associated disabilities and morbidities were also identified in children residing in Australasia (AAPC = 0.37, 95% CI = 0.12 to 0.69), Oceania (AAPC = 0.44, 95% CI = 0.23 to 0.67), and Western Europe (AAPC = 0.28, 95% CI = 0.19 to 0.39).

### 3.4. National

Different from global and regional analyses, the national results were based on age-standardized data (Table 2). Across nations, significant heterogenous changes in suboptimal breastfeeding mortalities and YLDs from 1990 to 2021 were identified and are presented in Supplementary Table S1. As indicated in regional results, many Latin American countries showed significant improvements in the burden of suboptimal breastfeeding as demonstrated by the reduced number of DALYs in countries, including Chile from 259.90 (95% CI = 198.26 to 320.82) to 14.60 (95% CI = 10.93 to 18.01), Colombia from 876.71 (95% CI = 657.79 to 1069.26) to 58.39 (95% CI = 34.84 to 88.81), El Salvador from 1857.17 (95% CI = 1424.59 to 2385.40) to 87.21 (95% CI = 50.14 to 133.63), and Mexico from 1701.14 (95% CI = 1386.93 to 2023.60) to 88.74 (95% CI = 57.97 to 126.64). Similarly, East Asian countries also showed significant improvements, including China, from a DALY of 914.87 (95% CI = 652.36 to 1184.19) to 36.63 (95% CI = 25.12 to 49.41).

## 4. Discussion

Overall, on a global level, significant improvements in suboptimal mortality and disease burden, including DALYs and YLDs, were identified from 1990 to 2021. However, considerable heterogeneity existed. When countries were stratified based on their SDI scores, those with high–middle SDI scores showed the most significant improvement, achieving a two-fold greater improvement in the AAPC than countries with low SDI scores. When countries were classified based on their geographical regions, the burden of suboptimal breastfeeding mortality and morbidity remained high in 2021 in low SDI countries in the Sub-Saharan Africa region. In contrast, countries with middle SDI scores in Tropical Latin American regions showed significant improvement in the burden of suboptimal breastfeeding, indicating success in large-scaled global and national initiatives to promote exclusive and continued breastfeeding.

The sociodemographic index (SDI) represents the social and economic development levels of a country, calculated based on the economy, education, and national fertility rate [17]. Our findings align with previous studies that used the Socio-Ecological Model to explain the complexity of multiple contributing factors that hinder optimal breastfeeding [8]. On an individual level, sociodemographic factors such as maternal education, household income, and maternal employment status have been identified to influence breastfeeding. On a structural level, pro-breastfeeding national policies such as a protected 6-to-12-month maternity leave and breastfeeding-friendly workplace policies support breastfeeding mothers to encourage breastfeeding. Furthermore, the availability of breastmilk substitutes is another structural factor that may influence breastfeeding. As these factors differ across nations of different wealth indices, significant heterogeneity in prevalence and improvements in suboptimal breastfeeding disease burden and mortality has been observed [18,19].

Previous UNICEF reports have indicated complicated disparities in breastfeeding practices across different economic contexts. For instance, most newborns are breastfed in low-income countries, whereas around 21% of babies are never breastfed in high-income countries [20]. In some high-income countries such as Sweden and Uruguay, almost all babies are breastfed, but in others like Ireland, only around 55% of babies are breastfed [20]. Within high-income countries, breastfeeding is more prevalent among mothers from higher-income households compared to those from lower-income backgrounds [20]. In contrast, in low-and middle-income countries, most mothers who do not breastfeed are from wealthier households [2,20]. In line with previous reports that identified significantly lower breastfeeding prevalence in high-income countries, our study found that mortalities and morbidities associated with suboptimal breastfeeding in high-income countries in the Australasia, Oceania, and Western European regions have significantly increased from 1990 to 2021. In contrast, significant improvements have been identified amongst most countries in Latin America.

At a national level, associations between household SES and breastfeeding are especially complex and often contradictory. A study in Uganda identified that residing in rural areas, as a sociodemographic factor, is positively associated with exclusive breastfeeding [19]. This suggests that despite relative economic and educational advantages, mothers working in urban areas may have insufficient structural support for breastfeeding, while having better access to breastmilk substitutes, thereby reducing the breastfeeding rate [19]. However, this contrasts with an Ethiopian study that identified rural residency as a risk factor for the early cessation of breastfeeding [21]. Studies conducted in Ghana have also identified a drastic decrease in exclusive breastfeeding from 63% in 2008 to 43% in 2018, citing early introductions of supplementary food preparations and the indiscriminate advertisement of breastmilk substitutes as barriers in achieving exclusive breastfeeding practices [22].

The difference in national results highlights the complexity of the interactions between individual demographic factors and the structural factors of national policy that influence the availability of breastmilk substitutes and supportive interventions and environments for breastfeeding. While household income, SES, and maternal education act as individual-level factors on the socioecological resilience model that predispose mothers to suboptimal breastfeeding, the structural factors of interventions and national policies may help alleviate some risks [18,23]. Pro-breastfeeding national policies aid mothers in achieving optimal breastfeeding through fostering a supportive environment to encourage prolonged breastfeeding to up to 2 years of age and exclusive breastfeeding in the first 6 months [24,25,26]. In contrast, a lack of supportive national policies leaves vulnerable mothers with a low education and poor SES at an increased risk of suboptimal breastfeeding.

Availability and accessibility to breastmilk substitutes further influence maternal breastfeeding practices. The individual sociodemographic factors of household income and maternal employment may influence breastmilk substitute use, while structural factors of rural residency and national policies against advertisements of breastmilk substitutes can influence the availability of breastmilk substitutes. In low-income countries or countries with low SDI, breastmilk substitutes may not be available for poorer mothers in rural regions due to financial and geographical barriers, and poor individual sociodemographic factors such as low education and a lack of health literacy may be bigger factors that are hindering exclusive and continued breastfeeding. Wealthier mothers in low-income countries with access to breastmilk substitutes or employed mothers in urban regions without established policies to support continued breastfeeding are at a greater risk of suboptimal breastfeeding. The problem is reversed in high-income countries where wealthier and employed mothers with better sociodemographic factors are more likely to achieve optimal breastfeeding due to support from established policies. However, mothers in high-income countries with a lower SES are at a greater risk of suboptimal breastfeeding due to poor education and worse access to healthcare and breastfeeding supports [23]. Thus, individual and structural factors influence both breastmilk substitute use and breastfeeding practices.

Significant wealth and health inequalities within high-income countries and a lack of government initiatives in low-income countries appear to drive significant differences in breastfeeding practices. Each vulnerable population must be addressed to reduce suboptimal breastfeeding. The contrasting influence of sociodemographic factors on exclusive breastfeeding and the early cessation of breastfeeding across nations suggest the need for more national surveillance and research to develop tailored policies and campaigns to address barriers specific to each nation. As cultural factors significantly influence food preferences and feeding practices, a single approach or intervention will not be able to effectively achieve optimal breastfeeding across all nations globally. Nonetheless, significant demographic risk factors including poor nutritional education and the indiscriminate advertising of breastmilk substitutes must be addressed across all nations to improve maternal health literacy to promote exclusive breastfeeding.

A balance of better individual sociodemographic factors with structural support from government policies to prevent the promotion of breastmilk substitutes and support continued breastfeeding through maternity leave and available breastfeeding facilities is required to reach optimal breastfeeding. Based on the risk factors identified, international codes aimed to promote breastfeeding have been developed, including the Code of Marketing of Breastmilk Substitutes and the ILO Maternity Protection Convention. The adoption of international codes is required to support employed mothers to continue breastfeeding when they return to work, while avoiding breastmilk substitutes when possible. To improve health literacy for breastfeeding amongst mothers, education and encouragement for continued and exclusive breastfeeding following birth are also critical to promote breastfeeding [24].

Different to previous studies where exclusive breastfeeding and continued breastfeeding to two years of age were assessed separately, suboptimal breastfeeding is defined as a combination of both non-exclusive breastfeeding until 6 months of age and cessation of breastfeeding before 2 years of age. The more stringent criteria for defining suboptimal breastfeeding may explain why less improvements have been identified in the current study. When a stricter criterion was applied for the assessment of breastfeeding, we were able to identify more nations in both high and low SDIs that require further support in achieving optimal breastfeeding. The identification of such trends in relation to wealth and health inequalities between countries with different SDI scores and within high SDI nations suggests the need for nation-specific policies that account for different cultural practices and economic conditions to achieve optimal breastfeeding.

The success of implementing pro-breastfeeding policies have been demonstrated in different Latin American countries, which aligns with the current study that identified the most significant improvements in suboptimal breastfeeding morbidity and mortalities in Latin American regions. In Brazil, various pro-breastfeeding policies have been introduced since 1981, including the extension of maternity leave to 6 months, the establishment of the Human Milk Banks network in 2004 to help promote, protect, and support breastfeeding collection and storage, and the Breastfeeding and Feeding Brazil Strategy in 2012 that aims to improve counseling to support and promote feeding infant and young children. Extensive national efforts in Brazil have significantly improved the prevalence of exclusive breastfeeding and increased the breastfeeding duration [25,26]. Nonetheless, policy implementation for breastfeeding is complex given the influence of economic resources, cultural norms, and political will.

The authors acknowledge that GBD data are a model-based estimates with data extracted from various vital registration systems, verbal autopsies, censuses, household surveys, disease-specific registers, health service contact data, and other sources. Thus, the inherent limitations must be recognized, including sparse, missing, or conflicting data for specific locations, differences in case definitions across the different data extracted, and difficulties in dealing with anomalous studies or data [27]. The use of various data sources itself may also create bias [27]. Nonetheless, the GBD has constantly reviewed their methodology addressing the statistical challenges identified [28].

## 5. Conclusions

Significant global improvements in suboptimal breastfeeding mortality and morbidity have occurred in the last 30 years. However, the extent of improvement differs significantly across nations, while some countries also showed no improvements or increased suboptimal breastfeeding mortality and disease burden. Compared to middle-income countries, mothers in low- and high-income countries require more support to improve optimal breastfeeding. The main barriers for suboptimal breastfeeding can be alleviated with policies that are in line with established global codes, but national policies must address environmental and individual factors specific to the sociodemographic factors of the mothers in each country. Within nations, mothers with different demographic factors also require different types of support to achieve optimal breastfeeding. In low-income countries, employed mothers may require more support to continue breastfeeding through policies focused on better maternal leave policies. In contrast, to improve breastfeeding in high-income countries, programs that target poor sociodemographic factors are required for mothers from a lower SES.

## Figures and Tables

**Table 1:** Under 5 years suboptimal breastfeeding DALYs, mortality, and YLDs across countries of different sociodemographic indices.

	DALYs ^1^						Mortalities						YLDs ^2^					
	1990	2021	AAPC ^3^ (95% CI ^4^)	1990	2021	AAPC (95% CI)	1990	2021	AAPC (95% CI)	1990	2021	AAPC (95% CI)	1990	2021	AAPC	1990	2021	AAPC (95% CI)
	Rate	Rate		Number	Number		Rate	Rate		Number	Number	Rate	Rate	Rate		Number	Number	Rate
Global	7493.66	1391.89	−0.82 (−0.85, −0.78)	47,368,654.78	9,161,024.86	−0.81 (−0.84, −0.76)	84.23	15.35	−0.82 (−0.85, −0.78)	532,453.25	101,023.15	−0.81 (−0.84, −0.76)	44.84	14.71	−0.6 (−0.63, −0.57)	283,438.12	96,845.1	−0.58 (−0.61, −0.55)
High SDI	206.84	55.18	−0.78 (−0.81, −0.74)	119,226.91	29,712.3	−0.81 (−0.84, −0.77)	2.01	0.49	−0.81 (−0.84, −0.78)	1158.91	265.06	−0.83 (−0.86, −0.81)	29.1	11.01	−0.31 (−0.37, −0.25)	16,775.75	5930.56	−0.39 (−0.45, −0.34)
High–middle SDI	2414.48	150.89	−0.92 (−0.94, −0.9)	2,523,277.81	105,688.91	−0.94 (−0.95, −0.93)	26.83	1.61	−0.92 (−0.94, −0.9)	28,043.47	1128.24	−0.94 (−0.95, −0.93)	41.34	6.32	−0.78 (−0.8, −0.76)	43,202.94	4428.47	−0.83 (−0.85, −0.82)
Middle SDI	5908.91	612.07	−0.89 (−0.91, −0.86)	12,103,370.6	1,081,028.22	−0.9 (−0.92, −0.88)	66.29	6.7	−0.89 (−0.91, −0.86)	135,778.97	11,836.11	−0.9 (−0.92, −0.88)	47.33	10.77	−0.73 (−0.75, −0.71)	96,955.83	19,015.58	−0.76 (−0.78, −0.75)
Low–middle SDI	10,712.25	1509.13	−0.87 (−0.9, −0.84)	18,018,609.29	2,891,162.01	−0.86 (−0.89, −0.82)	120.61	16.62	−0.87 (−0.9, −0.84)	202,867.48	31,831.85	−0.86 (−0.89, −0.83)	45.64	18.01	−0.56 (−0.6, −0.52)	76,770.74	34,512.35	−0.52 (−0.56, −0.47)
Low SDI	15,091.25	3046.93	−0.8 (−0.84, −0.75)	14,574,421.08	5,044,966.15	−0.63 (−0.71, −0.54)	170.1	33.74	−0.8 (−0.84, −0.75)	164,270.38	55,868.35	−0.63 (−0.71, −0.55)	51.28	19.86	−0.58 (−0.61, −0.53)	49,522.23	32,881.08	−0.23 (−0.29, −0.14)

^1^ Disability-adjusted life years. ^2^ Years lost to disability. ^3^ Average annual percentage change. ^4^ Confidence interval.

**Table 2:** Under 5 suboptimal breastfeeding DALYs, mortality, and YLDs across 21 geographical regions.

	DALYs ^1^						Mortality						YLDs ^2^					
	1990	2021	AAPC ^3^ (95% CI ^4^)	1990	2021	AAPC (95% CI)	1990	2021	AAPC (95% CI)	1990	2021	AAPC (95% CI)	1990	2021	AAPC (95% CI)	1990	2021	AAPC (95% CI)
	Rate	Rate		Number	Number		Rate	Rate		Number	Number		Rate	Rate		Number	Number	
Andean Latin America	6157.96	344.16	−0.94 (−0.96, −0.92)	335,398.76	21,186.1	−0.93 (−0.95, −0.91)	68.95	3.78	−0.94 (−0.96, −0.92)	3755.27	232.92	−0.93 (−0.95, −0.91)	64.64	4.79	−0.91 (−0.93, −0.9)	3520.88	295.1	−0.9 (−0.91, −0.89)
Australasia	80.95	26.64	−0.74 (−0.8, −0.66)	1248.83	483.78	−0.69 (−0.76, −0.6)	0.76	0.21	−0.8 (−0.85, −0.74)	11.77	3.89	−0.76 (−0.82, −0.69)	13.46	7.46	0.17 (−0.05, 0.44)	207.65	135.44	0.37 (0.12, 0.69)
Caribbean	10,859.59	3080.31	−0.69 (−0.78, −0.57)	449,488.73	119,151.75	−0.71 (−0.8, −0.6)	121.99	34.21	−0.69 (−0.78, −0.57)	5049.45	1323.21	−0.71 (−0.8, −0.6)	71.33	12.08	−0.66 (−0.7, −0.62)	2952.44	467.45	−0.69 (−0.72, −0.65)
Central Asia	8110.97	1098.18	−0.84 (−0.87, −0.8)	768,863.1	109,785.3	−0.83 (−0.86, −0.79)	91.13	12.17	−0.84 (−0.87, −0.8)	8638.89	1216.24	−0.83 (−0.86, −0.79)	49.3	5.71	−0.87 (−0.88, −0.85)	4673.73	570.92	−0.86 (−0.87, −0.84)
Central Europe	1163.02	213.85	−0.81 (−0.85, −0.77)	103,900.53	11,944.88	−0.89 (−0.91, −0.86)	12.69	2.34	−0.81 (−0.85, −0.77)	1133.97	130.95	−0.89 (−0.91, −0.86)	40.26	3.45	−0.68 (−0.7, −0.65)	3596.33	192.98	−0.8 (−0.82, −0.79)
Central Latin America	5588.09	485.52	−0.92 (−0.94, −0.89)	1,279,358.78	97,542.08	−0.93 (−0.95, −0.91)	62.63	5.37	−0.92 (−0.94, −0.89)	14,338.26	1079.76	−0.93 (−0.95, −0.91)	53.82	3.82	−0.87 (−0.88, −0.85)	12,322.29	767.78	−0.88 (−0.9, −0.87)
Central Sub-Saharan Africa	16,350.04	1544.68	−0.9 (−0.93, −0.86)	1,757,623.67	325,410.83	−0.8 (−0.86, −0.71)	184.23	17	−0.9 (−0.93, −0.86)	19,805.11	3580.34	−0.8 (−0.86, −0.71)	62.4	19.84	−0.65 (−0.7, −0.59)	6707.63	4179.27	−0.29 (−0.39, −0.17)
East Asia	4887.01	140.91	−0.96 (−0.97, −0.95)	5,856,276.32	112,830.93	−0.97 (−0.98, −0.96)	54.81	1.55	−0.96 (−0.97, −0.94)	65,684.81	1244.34	−0.97 (−0.98, −0.96)	39.95	1.4	−0.95 (−0.96, −0.94)	47,875.09	1124.01	−0.97 (−0.97, −0.96)
Eastern Europe	799.7	134.6	−0.84 (−0.87, −0.82)	137,776.53	13,619.94	−0.91 (−0.92, −0.9)	8.54	1.4	−0.85 (−0.87, −0.82)	1471.18	141.74	−0.91 (−0.92, −0.9)	43.92	8.87	−0.78 (−0.8, −0.76)	7566	897.72	−0.87 (−0.88, −0.86)
Eastern Sub-Saharan Africa	13,006.23	2121.74	−0.85 (−0.89, −0.79)	4,684,830.02	1,353,593.81	−0.73 (−0.8, −0.63)	146.55	23.46	−0.85 (−0.89, −0.79)	52,787.84	14,964.1	−0.73 (−0.8, −0.63)	50.58	17.37	−0.66 (−0.69, −0.61)	18,219.89	11,080.48	−0.39 (−0.45, −0.32)
High-Income Asia Pacific	110.68	38.29	−0.79 (−0.83, −0.73)	11,345.1	2470.41	−0.87 (−0.9, −0.83)	1.21	0.31	−0.83 (−0.87, −0.79)	124.23	19.71	−0.9 (−0.92, −0.86)	3.5	10.89	−0.21 (−0.32, −0.12)	358.46	702.74	−0.5 (−0.57, −0.44)
High-Income North America	117.28	40.95	−0.75 (−0.79, −0.71)	25,211.85	8393.38	−0.77 (−0.8, −0.73)	1.02	0.44	−0.74 (−0.78, −0.7)	218.85	89.31	−0.75 (−0.79, −0.71)	27.23	1.85	−0.88 (−0.89, −0.86)	5853.15	378.27	−0.88 (−0.9, −0.87)
North Africa and Middle East	7268.88	636.43	−0.9 (−0.92, −0.87)	3,877,351.15	389,093.75	−0.88 (−0.91, −0.85)	81.45	6.92	−0.9 (−0.92, −0.87)	43,448.23	4232.88	−0.88 (−0.91, −0.85)	64.07	15.09	−0.7 (−0.74, −0.65)	34,174.64	9225.34	−0.64 (−0.68, −0.59)
Oceania	4288.67	2210.15	−0.55 (−0.67, −0.38)	42,164.73	42,754.3	−0.14 (−0.36, 0.19)	47.98	24.42	−0.55 (−0.67, −0.39)	471.77	472.46	−0.14 (−0.37, 0.18)	48.45	20.75	−0.25 (−0.36, −0.14)	476.34	401.49	0.44 (0.23, 0.67)
South Asia	7476.08	1364	−0.87 (−0.9, −0.82)	12,094,148.3	2,163,224.14	−0.87 (−0.9, −0.82)	84.14	15.01	−0.87 (−0.9, −0.82)	136,117.95	23,799.33	−0.87 (−0.9, −0.82)	32.66	16.92	−0.47 (−0.51, −0.42)	52,834.88	26,832.32	−0.46 (−0.5, −0.41)
Southeast Asia	7687.73	952.71	−0.89 (−0.92, −0.86)	4,572,961.7	536,227.27	−0.9 (−0.92, −0.86)	86.35	10.37	−0.89 (−0.92, −0.86)	51,361.64	5837.11	−0.9 (−0.92, −0.86)	51.22	21.98	−0.57 (−0.6, −0.53)	30,468.09	12,372.6	−0.58 (−0.61, −0.55)
Southern Latin America	862.57	120.19	−0.89 (−0.91, −0.85)	44,502.05	5142.12	−0.9 (−0.93, −0.88)	9.43	1.27	−0.89 (−0.92, −0.85)	486.68	54.17	−0.91 (−0.93, −0.88)	28.59	6.59	−0.73 (−0.76, −0.69)	1474.82	281.99	−0.77 (−0.8, −0.74)
Southern Sub-Saharan Africa	10,117.1	3481.01	−0.7 (−0.76, −0.62)	725,792.52	279,494.2	−0.68 (−0.75, −0.59)	113.77	38.53	−0.7 (−0.77, −0.62)	8161.72	3093.78	−0.68 (−0.75, −0.59)	58.48	25.32	−0.59 (−0.62, −0.55)	4195.5	2032.87	−0.55 (−0.6, −0.51)
Tropical Latin America	10,418.53	239.76	−0.97 (−0.98, −0.96)	1,828,996.76	41,256.33	−0.97 (−0.98, −0.96)	117.13	2.6	−0.97 (−0.98, −0.96)	20,561.64	447.27	−0.97 (−0.98, −0.96)	63.67	6.63	−0.88 (−0.9, −0.86)	11,177.75	1141.03	−0.88 (−0.9, −0.86)
Western Europe	118.82	58.6	−0.52 (−0.61, −0.41)	27,280.25	12,439.56	−0.55 (−0.64, −0.46)	0.87	0.37	−0.67 (−0.73, −0.6)	199.13	79.6	−0.7 (−0.75, −0.63)	42.12	24.97	0.39 (0.28, 0.51)	9669.37	5300.24	0.28 (0.19, 0.39)
Western Sub-Saharan Africa	24,511.9	4396.03	−0.76 (−0.81, −0.7)	8,744,135.08	3,514,980.01	−0.46 (−0.57, −0.33)	276.47	48.75	−0.76 (−0.81, −0.7)	98,624.85	38,980.03	−0.46 (−0.57, −0.33)	70.4	23.09	−0.61 (−0.65, −0.56)	25,113.18	18,465.06	−0.13 (−0.21, −0.02)

^1^ Disability-adjusted life years. ^2^ Years lost to disability. ^3^ Average annual percentage change. ^4^ Confidence interval.

## Data Availability

Data analyzed will be made available on reasonable request to corresponding authors.

## Supplementary Materials

The following supporting information can be downloaded at: https://www.mdpi.com/article/10.3390/nu17071134/s1, Table S1: National changes in suboptimal breastfeeding DALYs, mortalities and YLDs.

## Abbreviations

The following abbreviations are used in this manuscript:

LMICs	Low-to-middle income countries
WHO	World Health Organization
DALYs	Disability-Adjusted Life Years
YLDs	Years Lost to Disability
AAPC	Average Annual Percentage Change
ASR	Age-Standardized Rate
SDI	Sociodemographic Index Score
ILO	International Labor Organization
BMS	Breastmilk Substitutes
WHA	World Health Assembly
LDI	Lag-Distributed Income
CI	Confidence Interval
