# Supplementary material for: Trend and Burden of Suboptimal Breastfeeding in Children Under Five Years of Age in 1990–2021: A Systematic Analysis for the Global Burden of Disease Study 2021
Source: Nutrients. 2025 Mar 25;17(7):1134. doi: 10.3390/nu17071134 (PMC11990223; doi:10.3390/nu17071134)
Supplement: Supplementary file 1 [file nutrients-17-01134-s001.zip › nutrients-3523924-supplementary.pdf]

Supplementary Table S1: National changes in suboptimal breastfeeding DALYs, mortalities and YLDs

	DALYs		Mortality		YLDs	
	1990	2021	1990	2021	1990	2021
Afghanistan	3646.06 (2302.65, 5511.65)	490.98 (309.30, 704.21)	40.52 (61.28, 25.57)	5.44 (7.81, 3.42)	9.20 (14.08, 5.57)	2.74 (4.10, 1.78)
Albania	879.76 (559.88, 1278.54)	47.55 (26.54, 76.37)	9.76 (14.20, 6.20)	0.52 (0.84, 0.29)	3.59 (5.40, 2.22)	0.79 (1.18, 0.49)
Algeria	545.41 (359.87, 789.17)	54.99 (35.08, 77.28)	5.92 (8.63, 3.86)	0.59 (0.84, 0.37)	14.16 (21.28, 8.60)	2.14 (3.15, 1.31)
American Samoa	167.63 (110.94, 235.21)	67.79 (42.01, 97.85)	1.84 (2.59, 1.21)	0.74 (1.08, 0.46)	2.38 (3.72, 1.43)	1.38 (2.13, 0.79)
Andorra	14.92 (9.87, 21.02)	4.16 (2.23, 6.90)	0.12 (0.18, 0.07)	0.01 (0.01, 0.00)	4.34 (7.48, 2.24)	3.70 (6.41, 1.87)
Angola	6530.49 (4347.38, 9097.79)	399.32 (255.32, 558.01)	72.59 (101.17, 48.29)	4.41 (6.18, 2.81)	18.08 (26.17, 11.86)	4.12 (6.23, 2.67)
Antigua and Barbuda	152.89 (115.95, 192.70)	63.60 (45.15, 86.13)	1.68 (2.12, 1.27)	0.70 (0.95, 0.49)	2.39 (3.51, 1.52)	0.91 (1.40, 0.58)
Argentina	274.77 (219.13, 328.13)	35.14 (24.33, 47.91)	2.98 (3.57, 2.38)	0.38 (0.53, 0.27)	7.22 (10.82, 4.38)	0.66 (1.02, 0.40)
Armenia	1137.64 (855.20, 1429.77)	115.34 (75.11, 160.95)	12.53 (15.79, 9.40)	1.27 (1.78, 0.82)	13.07 (19.34, 8.36)	1.10 (1.67, 0.67)
Australia	22.25 (17.18, 27.36)	4.42 (3.25, 5.73)	0.23 (0.28, 0.18)	0.04 (0.06, 0.03)	1.63 (2.79, 0.87)	0.48 (0.79, 0.27)
Austria	26.28 (20.05, 33.63)	7.35 (5.21, 10.09)	0.22 (0.27, 0.17)	0.04 (0.05, 0.03)	6.94 (11.72, 3.13)	3.72 (6.02, 1.96)
Azerbaijan	3078.04 (2213.89, 3979.75)	504.34 (346.04, 693.10)	34.12 (44.16, 24.51)	5.59 (7.70, 3.83)	15.00 (21.91, 9.59)	2.11 (3.12, 1.28)
Bahamas	277.72 (202.83, 368.05)	61.44 (40.81, 87.62)	3.07 (4.08, 2.24)	0.68 (0.97, 0.45)	1.93 (2.98, 1.21)	0.45 (0.70, 0.28)
Bahrain	251.97 (183.45, 333.56)	26.45 (18.49, 36.41)	2.78 (3.69, 2.00)	0.29 (0.40, 0.20)	2.28 (3.65, 1.38)	0.82 (1.28, 0.47)
Bangladesh	2192.45 (1390.84, 3086.91)	146.90 (83.66, 228.11)	24.33 (34.29, 15.41)	1.63 (2.54, 0.93)	8.25 (12.46, 5.11)	0.58 (0.85, 0.35)
Barbados	162.23 (125.03, 205.73)	48.20 (30.36, 71.22)	1.78 (2.27, 1.36)	0.53 (0.78, 0.33)	2.31 (3.52, 1.46)	0.94 (1.42, 0.55)
Belarus	193.34 (142.71, 243.26)	23.32 (17.09, 30.45)	2.04 (2.61, 1.49)	0.22 (0.30, 0.16)	9.95 (14.62, 6.36)	3.15 (4.79, 1.88)
Belgium	39.48 (31.57, 47.55)	23.21 (17.17, 29.50)	0.41 (0.49, 0.33)	0.23 (0.30, 0.17)	2.94 (5.10, 1.43)	2.64 (4.24, 1.44)
Belize	1054.46 (873.26, 1264.97)	135.66 (96.03, 174.62)	11.69 (14.02, 9.66)	1.50 (1.93, 1.06)	6.14 (9.01, 3.92)	1.20 (1.82, 0.75)
Benin	2836.61 (2006.35, 3901.91)	450.81 (254.62, 735.63)	31.49 (43.38, 22.23)	5.00 (8.17, 2.82)	11.18 (16.45, 7.18)	2.39 (3.60, 1.54)
Bermuda	67.92 (51.76, 86.82)	9.80 (6.13, 13.65)	0.74 (0.95, 0.56)	0.10 (0.14, 0.06)	1.59 (2.43, 0.98)	1.14 (1.80, 0.65)
Bhutan	3327.91 (1678.05, 5159.95)	252.28 (138.41, 411.51)	36.99 (57.39, 18.62)	2.80 (4.57, 1.53)	6.51 (9.75, 4.16)	0.94 (1.45, 0.59)
Bolivia (Plurinational State of)	2189.78 (1434.51, 2944.37)	195.35 (126.28, 274.54)	24.32 (32.71, 15.92)	2.17 (3.06, 1.40)	9.25 (15.26, 5.10)	0.50 (0.83, 0.28)
Bosnia and Herzegovina	76.41 (56.08, 97.48)	32.63 (22.42, 47.45)	0.81 (1.04, 0.59)	0.35 (0.51, 0.24)	3.79 (5.68, 2.40)	1.45 (2.21, 0.88)
Botswana	1720.50 (1245.03, 2253.20)	787.81 (513.74, 1115.61)	19.05 (24.98, 13.77)	8.74 (12.39, 5.68)	12.83 (19.35, 7.93)	4.26 (6.52, 2.67)
Brazil	2324.91 (1937.70, 2758.59)	59.83 (42.76, 77.81)	25.79 (30.61, 21.49)	0.65 (0.85, 0.46)	11.17 (18.22, 6.37)	1.25 (2.09, 0.64)
Brunei Darussalam	39.52 (26.21, 55.39)	20.59 (13.70, 29.24)	0.43 (0.61, 0.29)	0.23 (0.32, 0.15)	0.48 (0.77, 0.27)	0.25 (0.41, 0.14)

Bulgaria	351.40 (275.66, 432.72)	89.91 (67.25, 113.03)	3.88 (4.79, 3.04)	0.99 (1.24, 0.74)	2.72 (3.94, 1.79)	1.11 (1.68, 0.68)
Burkina Faso	5228.00 (3427.69, 7135.56)	1110.92 (709.51, 1598.97)	58.08 (79.30, 38.07)	12.35 (17.78, 7.87)	15.29 (22.10, 9.67)	3.13 (4.70, 1.93)
Burundi	1569.33 (900.84, 2394.49)	249.21 (115.03, 450.68)	17.41 (26.60, 9.98)	2.75 (5.00, 1.26)	7.73 (11.72, 4.63)	2.28 (3.54, 1.39)
Cabo Verde	1721.61 (1225.69, 2344.95)	139.74 (80.24, 224.83)	19.03 (25.95, 13.51)	1.55 (2.49, 0.88)	15.44 (23.34, 9.60)	1.18 (1.77, 0.73)
Cambodia	4633.13 (3002.68, 6485.13)	290.22 (176.23, 430.57)	51.44 (72.05, 33.29)	3.19 (4.76, 1.94)	14.71 (21.41, 9.27)	3.39 (5.17, 2.12)
Cameroon	2593.66 (1716.12, 3683.10)	620.62 (352.54, 1051.37)	28.74 (40.86, 18.97)	6.87 (11.68, 3.87)	15.94 (23.65, 9.96)	4.51 (6.69, 2.88)
Canada	23.35 (18.72, 28.26)	13.99 (10.92, 17.67)	0.23 (0.28, 0.18)	0.15 (0.19, 0.12)	2.77 (4.65, 1.42)	0.61 (0.98, 0.35)
Central African Republic	6378.63 (4225.57, 8502.47)	1733.44 (1084.23, 2605.54)	70.88 (94.58, 46.86)	19.26 (29.00, 12.04)	16.42 (23.54, 10.75)	5.17 (7.84, 3.28)
Chad	6829.77 (4837.71, 9622.55)	3236.40 (2071.36, 4981.79)	75.96 (107.04, 53.82)	35.99 (55.43, 22.99)	15.61 (23.01, 9.81)	7.63 (11.09, 4.85)
Chile	259.90 (198.26, 320.82)	14.60 (10.93, 18.01)	2.86 (3.54, 2.18)	0.16 (0.19, 0.12)	3.56 (5.56, 2.11)	0.64 (1.05, 0.35)
China	914.87 (652.36, 1184.19)	36.63 (25.12, 49.41)	10.11 (13.10, 7.20)	0.40 (0.55, 0.28)	7.77 (12.50, 4.41)	0.28 (0.46, 0.15)
Colombia	876.71 (657.79, 1069.26)	58.39 (34.84, 88.81)	9.70 (11.85, 7.27)	0.64 (0.98, 0.38)	6.60 (10.22, 3.75)	0.75 (1.23, 0.37)
Comoros	3246.38 (2176.75, 4515.15)	520.59 (319.66, 777.05)	35.97 (50.16, 24.04)	5.74 (8.60, 3.49)	17.48 (25.46, 11.21)	5.78 (8.46, 3.63)
Congo	2874.83 (1838.61, 4135.82)	387.66 (191.40, 751.19)	31.85 (45.94, 20.29)	4.27 (8.33, 2.08)	17.79 (26.57, 11.26)	4.88 (7.20, 3.05)
Cook Islands	202.74 (120.42, 302.47)	35.71 (21.65, 52.12)	2.24 (3.35, 1.32)	0.38 (0.57, 0.23)	1.82 (2.86, 1.04)	1.22 (2.09, 0.68)
Costa Rica	260.87 (203.41, 324.30)	30.83 (21.40, 40.98)	2.88 (3.58, 2.24)	0.34 (0.45, 0.23)	2.79 (4.56, 1.41)	0.69 (1.16, 0.35)
Croatia	88.82 (71.16, 107.75)	46.62 (32.98, 62.04)	0.97 (1.18, 0.78)	0.51 (0.68, 0.36)	1.56 (2.42, 0.95)	1.11 (1.69, 0.68)
Cuba	225.74 (185.55, 263.75)	42.47 (32.28, 54.23)	2.49 (2.91, 2.05)	0.46 (0.59, 0.35)	2.48 (3.75, 1.48)	0.90 (1.40, 0.51)
Cyprus	65.93 (43.15, 92.88)	12.13 (7.37, 17.70)	0.70 (0.99, 0.45)	0.09 (0.14, 0.05)	3.46 (6.01, 1.79)	4.01 (6.94, 1.97)
Czechia	140.27 (113.71, 169.82)	71.52 (54.46, 91.00)	1.55 (1.88, 1.26)	0.79 (1.00, 0.60)	0.84 (1.36, 0.50)	1.00 (1.53, 0.61)
Cote d'Ivoire	3572.14 (2391.19, 4994.64)	672.89 (408.14, 1040.83)	39.65 (55.51, 26.52)	7.46 (11.56, 4.52)	13.43 (19.58, 8.43)	3.13 (4.67, 1.88)
Democratic People's Republic of Korea	256.59 (157.55, 388.38)	41.58 (21.35, 65.76)	2.80 (4.26, 1.68)	0.43 (0.69, 0.20)	5.45 (8.53, 3.02)	3.14 (5.01, 1.89)
Democratic Republic of the Congo	3319.22 (2197.13, 4708.80)	320.81 (178.38, 573.91)	36.83 (52.28, 24.33)	3.54 (6.36, 1.96)	13.78 (19.82, 9.19)	3.48 (5.41, 2.20)
Denmark	34.13 (27.29, 41.04)	16.11 (11.97, 21.21)	0.33 (0.40, 0.27)	0.12 (0.16, 0.09)	4.35 (7.12, 2.46)	5.15 (8.38, 3.05)
Djibouti	3152.18 (2061.01, 4434.25)	369.86 (203.17, 594.06)	34.96 (49.22, 22.85)	4.09 (6.59, 2.24)	16.57 (24.71, 10.19)	2.82 (4.16, 1.72)
Dominica	225.91 (161.81, 305.46)	162.88 (101.16, 254.14)	2.49 (3.37, 1.78)	1.81 (2.82, 1.12)	2.08 (3.17, 1.23)	0.89 (1.36, 0.55)
Dominican Republic	1730.34 (1263.99, 2293.98)	256.34 (138.37, 384.04)	19.15 (25.43, 13.96)	2.83 (4.26, 1.52)	14.52 (21.64, 8.78)	2.31 (3.56, 1.42)
Ecuador	1074.97 (847.64, 1318.87)	61.59 (37.73, 92.13)	11.82 (14.48, 9.31)	0.68 (1.02, 0.41)	15.51 (24.25, 8.97)	0.44 (0.70, 0.25)
Egypt	2381.66 (1621.17, 3294.89)	233.70 (147.36, 328.75)	26.38 (36.52, 17.90)	2.54 (3.60, 1.60)	14.89 (22.05, 9.10)	5.76 (8.38, 3.61)
El Salvador	1857.17 (1424.59, 2385.40)	87.21 (50.14, 133.63)	20.64 (26.53, 15.82)	0.97 (1.48, 0.55)	6.59 (10.50, 3.55)	0.47 (0.74, 0.26)

Equatorial Guinea	5616.38 (3444.81, 7850.04)	218.71 (126.25, 350.98)	62.41 (87.30, 38.26)	2.41 (3.88, 1.38)	17.84 (26.05, 11.37)	2.59 (3.93, 1.69)
Eritrea	2909.00 (1872.09, 4178.03)	346.03 (192.56, 544.94)	32.31 (46.44, 20.77)	3.83 (6.04, 2.12)	11.24 (17.23, 6.78)	2.81 (4.36, 1.63)
Estonia	129.29 (100.33, 158.51)	18.73 (14.68, 23.55)	1.37 (1.69, 1.06)	0.16 (0.20, 0.12)	6.09 (9.36, 3.83)	4.50 (7.07, 2.73)
Eswatini	3294.85 (2517.18, 4107.41)	1050.75 (677.43, 1463.25)	36.57 (45.65, 27.94)	11.66 (16.25, 7.50)	15.10 (22.60, 9.46)	5.17 (7.72, 3.27)
Ethiopia	3020.45 (1801.63, 4381.90)	334.87 (214.77, 480.37)	33.54 (48.70, 19.98)	3.69 (5.31, 2.36)	11.07 (16.84, 6.73)	3.44 (5.23, 1.99)
Fiji	275.17 (179.41, 388.32)	104.89 (61.90, 161.37)	3.02 (4.28, 1.96)	1.14 (1.76, 0.66)	4.67 (7.26, 2.86)	3.18 (4.85, 1.89)
Finland	18.26 (14.14, 22.78)	7.68 (5.57, 10.13)	0.16 (0.20, 0.12)	0.05 (0.07, 0.04)	4.22 (6.88, 2.26)	2.80 (4.74, 1.48)
France	34.61 (28.26, 40.98)	16.40 (12.01, 21.54)	0.33 (0.39, 0.28)	0.12 (0.15, 0.09)	4.97 (7.98, 2.80)	5.62 (9.34, 3.05)
Gabon	1941.39 (1063.44, 3216.17)	247.99 (108.57, 471.36)	21.48 (35.70, 11.65)	2.72 (5.20, 1.17)	15.23 (23.38, 9.17)	3.62 (5.56, 2.24)
Gambia	1850.73 (1212.02, 2552.97)	232.13 (148.34, 343.30)	20.53 (28.36, 13.43)	2.58 (3.81, 1.64)	8.49 (12.90, 5.35)	1.15 (1.71, 0.71)
Georgia	910.61 (678.58, 1133.50)	38.01 (26.59, 51.73)	10.04 (12.50, 7.45)	0.41 (0.57, 0.29)	9.81 (14.79, 6.29)	0.89 (1.40, 0.54)
Germany	23.81 (18.74, 29.19)	9.59 (7.25, 12.51)	0.23 (0.29, 0.18)	0.08 (0.10, 0.06)	3.20 (5.50, 1.55)	2.68 (4.45, 1.42)
Ghana	1753.88 (1107.08, 2517.99)	199.20 (117.32, 310.20)	19.38 (27.89, 12.17)	2.20 (3.43, 1.29)	15.15 (21.77, 9.62)	1.89 (2.78, 1.20)
Greece	17.81 (13.74, 22.26)	9.76 (6.86, 12.59)	0.15 (0.19, 0.12)	0.07 (0.10, 0.05)	4.09 (7.19, 2.06)	3.13 (5.33, 1.59)
Greenland	143.13 (95.42, 197.08)	19.19 (13.05, 27.03)	1.58 (2.18, 1.06)	0.21 (0.30, 0.14)	0.87 (1.41, 0.50)	0.21 (0.35, 0.12)
Grenada	282.18 (200.61, 372.08)	64.86 (42.78, 89.54)	3.12 (4.12, 2.21)	0.72 (0.99, 0.47)	2.29 (3.42, 1.40)	0.63 (0.99, 0.39)
Guam	98.09 (64.70, 136.61)	57.20 (37.44, 79.47)	1.07 (1.50, 0.70)	0.61 (0.86, 0.40)	1.96 (3.01, 1.20)	2.18 (3.31, 1.30)
Guatemala	2095.45 (1572.45, 2588.40)	376.58 (236.85, 524.74)	23.35 (28.85, 17.51)	4.19 (5.85, 2.64)	3.34 (5.55, 1.60)	0.86 (1.48, 0.41)
Guinea	5365.25 (3487.90, 7250.84)	733.35 (403.87, 1167.16)	59.64 (80.61, 38.73)	8.14 (12.97, 4.47)	13.23 (19.15, 8.22)	2.62 (3.79, 1.68)
Guinea-Bissau	3541.93 (2244.70, 4895.65)	347.39 (206.64, 537.79)	39.36 (54.46, 24.95)	3.85 (5.97, 2.28)	9.47 (13.63, 6.04)	1.78 (2.68, 1.08)
Guyana	1556.80 (1233.43, 1949.10)	196.47 (129.77, 284.37)	17.30 (21.65, 13.70)	2.18 (3.16, 1.44)	4.48 (6.54, 2.96)	0.93 (1.37, 0.58)
Haiti	7366.40 (5774.68, 9360.47)	1674.65 (1096.27, 2381.55)	82.00 (104.20, 64.28)	18.64 (26.51, 12.19)	8.88 (13.41, 5.56)	2.69 (4.17, 1.68)
Honduras	1921.64 (1405.44, 2470.99)	209.67 (135.12, 296.12)	21.39 (27.50, 15.64)	2.33 (3.29, 1.50)	3.39 (5.40, 1.85)	0.90 (1.39, 0.51)
Hungary	134.28 (109.29, 160.95)	85.02 (60.97, 109.39)	1.48 (1.77, 1.20)	0.93 (1.20, 0.67)	1.77 (3.03, 0.97)	1.30 (1.98, 0.78)
Iceland	17.17 (13.08, 21.60)	9.71 (6.86, 13.34)	0.14 (0.18, 0.11)	0.06 (0.08, 0.04)	4.30 (7.08, 2.22)	4.37 (7.31, 2.31)
India	2497.76 (1763.44, 3222.43)	334.35 (217.51, 472.19)	27.73 (35.79, 19.56)	3.69 (5.23, 2.39)	8.62 (13.27, 5.19)	3.25 (4.93, 1.98)
Indonesia	3318.80 (2268.16, 4309.72)	292.41 (199.26, 382.10)	36.83 (47.87, 25.12)	3.17 (4.16, 2.15)	13.16 (19.74, 8.43)	8.26 (12.58, 5.20)
Iran (Islamic Republic of)	474.11 (314.35, 735.98)	10.80 (7.23, 15.51)	5.18 (8.08, 3.43)	0.11 (0.16, 0.07)	8.96 (13.35, 5.36)	0.61 (0.96, 0.37)
Iraq	944.57 (633.02, 1325.61)	129.26 (84.69, 181.97)	10.37 (14.60, 6.86)	1.40 (1.99, 0.91)	14.23 (21.15, 8.81)	3.34 (4.92, 2.05)
Ireland	33.86 (26.17, 41.87)	12.13 (8.97, 15.93)	0.34 (0.43, 0.27)	0.09 (0.11, 0.06)	3.14 (5.51, 1.54)	4.23 (7.25, 2.22)

Israel	35.90 (28.56, 42.94)	13.61 (10.25, 17.58)	0.37 (0.45, 0.30)	0.13 (0.16, 0.10)	2.45 (4.30, 1.21)	2.11 (3.49, 1.15)
Italy	28.61 (22.73, 34.35)	10.63 (8.11, 13.35)	0.29 (0.35, 0.23)	0.10 (0.13, 0.07)	2.48 (4.31, 1.33)	1.59 (2.58, 0.89)
Jamaica	450.82 (363.41, 549.30)	57.27 (36.55, 84.05)	4.95 (6.04, 3.98)	0.63 (0.93, 0.40)	7.32 (11.03, 4.42)	0.90 (1.34, 0.57)
Japan	23.47 (18.34, 28.61)	9.46 (7.06, 12.27)	0.22 (0.27, 0.17)	0.07 (0.08, 0.05)	3.97 (6.51, 2.12)	3.39 (5.67, 1.84)
Jordan	407.66 (271.81, 576.93)	66.62 (47.29, 91.40)	4.45 (6.33, 2.94)	0.72 (1.00, 0.51)	8.59 (13.31, 5.47)	1.79 (2.68, 1.10)
Kazakhstan	1165.42 (846.84, 1490.55)	59.13 (34.94, 86.56)	12.86 (16.49, 9.33)	0.65 (0.96, 0.38)	10.83 (15.83, 6.77)	0.69 (1.05, 0.44)
Kenya	3279.01 (2277.29, 4233.90)	452.67 (317.91, 585.58)	36.38 (47.00, 25.20)	4.98 (6.45, 3.49)	14.28 (21.05, 9.07)	5.74 (8.48, 3.56)
Kiribati	1482.60 (979.72, 2152.98)	262.82 (157.85, 388.58)	16.46 (23.92, 10.86)	2.91 (4.30, 1.74)	9.79 (14.96, 5.86)	2.64 (4.12, 1.61)
Kuwait	122.60 (94.32, 149.38)	49.50 (34.61, 66.73)	1.28 (1.58, 0.98)	0.53 (0.72, 0.37)	7.40 (11.55, 4.44)	1.56 (2.40, 0.93)
Kyrgyzstan	1468.26 (1054.59, 1907.17)	87.50 (60.34, 122.50)	16.25 (21.16, 11.66)	0.96 (1.35, 0.66)	9.52 (14.06, 6.07)	1.17 (1.72, 0.75)
Lao People's Democratic Republic	8197.33 (5683.78, 11001.42)	684.88 (453.96, 947.20)	91.15 (122.42, 63.16)	7.56 (10.48, 4.99)	16.35 (24.06, 10.51)	6.42 (9.60, 3.96)
Latvia	90.48 (66.26, 116.07)	14.17 (10.28, 18.78)	0.95 (1.24, 0.70)	0.13 (0.18, 0.09)	4.73 (7.17, 3.00)	2.22 (3.42, 1.35)
Lebanon	490.76 (336.96, 687.33)	91.97 (58.89, 137.39)	5.40 (7.58, 3.69)	1.00 (1.50, 0.63)	6.31 (9.66, 3.83)	2.21 (3.38, 1.32)
Lesotho	3492.75 (2677.77, 4441.27)	1845.70 (1190.32, 2563.48)	38.77 (49.29, 29.72)	20.54 (28.54, 13.23)	14.11 (20.77, 8.97)	4.06 (6.14, 2.61)
Liberia	6494.32 (4445.41, 8977.38)	610.34 (318.79, 1062.22)	72.24 (99.93, 49.39)	6.77 (11.81, 3.51)	18.25 (26.81, 11.53)	3.70 (5.49, 2.27)
Libya	461.19 (277.44, 722.06)	41.65 (23.93, 63.34)	5.05 (7.95, 3.00)	0.45 (0.69, 0.25)	8.47 (12.73, 5.12)	1.20 (1.83, 0.72)
Lithuania	133.13 (107.88, 158.76)	31.18 (24.44, 38.40)	1.40 (1.68, 1.14)	0.30 (0.37, 0.23)	7.37 (11.46, 4.60)	4.38 (6.75, 2.69)
Luxembourg	23.96 (19.24, 29.48)	12.25 (9.00, 16.14)	0.23 (0.28, 0.18)	0.10 (0.13, 0.07)	3.66 (6.04, 2.03)	3.15 (5.23, 1.80)
Madagascar	4273.41 (3165.39, 5439.53)	942.45 (578.54, 1363.23)	47.46 (60.45, 35.12)	10.45 (15.15, 6.39)	15.86 (23.41, 10.10)	4.64 (6.90, 3.04)
Malawi	5979.29 (3672.53, 8317.93)	375.67 (239.55, 560.51)	66.47 (92.52, 40.76)	4.16 (6.22, 2.63)	17.36 (25.67, 11.39)	2.59 (3.87, 1.70)
Malaysia	201.22 (142.25, 275.27)	44.07 (29.39, 62.89)	2.20 (3.03, 1.55)	0.47 (0.67, 0.30)	3.86 (5.89, 2.35)	2.25 (3.51, 1.32)
Maldives	1549.55 (1095.68, 2052.20)	87.33 (57.94, 123.68)	17.12 (22.75, 12.08)	0.92 (1.32, 0.60)	14.54 (21.73, 8.85)	4.73 (7.10, 2.91)
Mali	3751.62 (2381.59, 5636.35)	573.46 (359.20, 841.86)	41.65 (62.65, 26.40)	6.36 (9.34, 3.97)	15.77 (23.42, 9.83)	3.08 (4.54, 1.98)
Malta	23.20 (18.02, 28.97)	10.07 (7.11, 13.34)	0.22 (0.29, 0.17)	0.08 (0.10, 0.05)	3.03 (5.34, 1.45)	3.10 (5.19, 1.53)
Marshall Islands	448.02 (297.38, 618.44)	168.38 (104.49, 247.62)	4.93 (6.84, 3.26)	1.86 (2.75, 1.15)	5.57 (8.78, 3.40)	1.56 (2.35, 0.95)
Mauritania	1972.42 (1275.29, 2832.19)	276.79 (153.08, 458.50)	21.86 (31.43, 14.10)	3.04 (5.08, 1.67)	11.79 (17.33, 7.28)	3.73 (5.57, 2.39)
Mauritius	217.11 (170.41, 264.39)	91.82 (65.29, 119.35)	2.27 (2.78, 1.77)	0.98 (1.27, 0.69)	13.50 (20.48, 8.39)	4.29 (6.51, 2.69)
Mexico	1701.14 (1386.93, 2023.60)	88.74 (57.97, 126.64)	18.89 (22.48, 15.39)	0.98 (1.40, 0.64)	7.70 (12.36, 4.12)	0.70 (1.16, 0.37)
Micronesia (Federated States of)	574.88 (389.46, 811.63)	110.02 (66.20, 162.77)	6.34 (8.97, 4.28)	1.21 (1.80, 0.72)	6.26 (9.85, 3.65)	1.33 (2.10, 0.77)
Monaco	16.03 (10.66, 23.34)	7.18 (4.59, 10.23)	0.13 (0.21, 0.08)	0.04 (0.06, 0.03)	4.33 (7.63, 2.35)	3.39 (5.77, 1.72)

Mongolia	1357.48 (824.00, 2018.93)	104.68 (55.53, 164.29)	15.05 (22.40, 9.11)	1.16 (1.82, 0.61)	6.05 (9.25, 3.57)	0.56 (0.86, 0.35)
Montenegro	88.52 (55.60, 131.11)	9.45 (5.86, 14.12)	0.96 (1.43, 0.59)	0.10 (0.15, 0.06)	2.36 (3.75, 1.45)	0.81 (1.28, 0.46)
Morocco	2706.21 (2001.32, 3463.07)	179.25 (114.38, 272.07)	29.96 (38.34, 22.16)	1.96 (2.99, 1.23)	18.18 (27.20, 11.10)	3.56 (5.44, 2.25)
Mozambique	4229.19 (2810.55, 5940.63)	486.76 (294.17, 749.59)	46.97 (66.07, 31.17)	5.40 (8.33, 3.26)	15.47 (22.37, 10.12)	2.05 (3.01, 1.30)
Myanmar	4724.21 (2805.06, 6889.29)	464.53 (302.13, 666.93)	52.46 (76.59, 31.10)	5.10 (7.36, 3.28)	14.37 (20.55, 9.34)	6.59 (9.58, 4.04)
Namibia	2451.25 (1832.24, 3143.17)	789.68 (504.42, 1082.06)	27.19 (34.90, 20.32)	8.77 (12.04, 5.58)	12.96 (20.30, 7.81)	3.88 (5.90, 2.39)
Nauru	357.86 (231.01, 519.11)	164.20 (101.50, 236.99)	3.96 (5.75, 2.55)	1.81 (2.62, 1.11)	2.82 (4.50, 1.64)	1.80 (2.80, 1.03)
Nepal	2453.99 (1648.69, 3321.98)	186.02 (107.48, 287.60)	27.24 (36.90, 18.30)	2.06 (3.19, 1.19)	8.27 (12.28, 4.97)	1.04 (1.53, 0.65)
Netherlands	24.17 (19.06, 29.48)	16.40 (11.80, 21.48)	0.22 (0.27, 0.17)	0.11 (0.14, 0.08)	4.47 (7.80, 2.22)	6.56 (11.10, 3.29)
New Zealand	43.39 (33.52, 54.47)	11.36 (8.61, 14.19)	0.46 (0.59, 0.35)	0.11 (0.14, 0.08)	2.05 (3.48, 1.07)	1.46 (2.39, 0.78)
Nicaragua	2918.91 (2151.82, 3819.30)	158.94 (100.29, 231.81)	32.44 (42.49, 23.90)	1.76 (2.58, 1.11)	10.81 (17.54, 5.66)	0.75 (1.20, 0.41)
Niger	7084.31 (5020.69, 9530.22)	1178.50 (766.60, 1728.11)	78.79 (106.10, 55.82)	13.08 (19.21, 8.49)	17.82 (25.81, 11.42)	5.23 (7.61, 3.29)
Nigeria	5605.61 (4100.01, 7043.82)	1436.17 (991.16, 1924.05)	62.32 (78.39, 45.55)	15.94 (21.36, 10.99)	17.11 (25.26, 10.97)	6.44 (9.73, 3.93)
Niue	202.15 (128.27, 288.10)	319.35 (211.57, 452.24)	2.23 (3.19, 1.41)	3.54 (5.02, 2.34)	2.46 (3.87, 1.40)	1.77 (2.92, 1.03)
North Macedonia	860.72 (646.98, 1157.73)	73.34 (49.67, 102.82)	9.45 (12.72, 7.04)	0.80 (1.13, 0.54)	13.15 (18.97, 8.73)	1.36 (2.04, 0.85)
Northern Mariana Islands	78.06 (50.92, 114.85)	26.10 (17.36, 37.16)	0.84 (1.25, 0.54)	0.27 (0.39, 0.17)	2.42 (3.82, 1.38)	1.67 (2.63, 1.00)
Norway	10.54 (7.89, 13.35)	3.91 (2.64, 5.52)	0.10 (0.12, 0.07)	0.02 (0.03, 0.01)	1.96 (3.41, 1.00)	2.15 (3.60, 1.21)
Oman	267.48 (159.78, 396.16)	38.96 (24.62, 55.92)	2.92 (4.36, 1.71)	0.42 (0.61, 0.27)	5.62 (8.73, 3.39)	1.01 (1.52, 0.61)
Pakistan	4084.12 (3035.34, 5202.99)	533.61 (317.83, 812.04)	45.34 (57.85, 33.71)	5.90 (9.00, 3.51)	14.95 (22.73, 9.55)	4.15 (6.46, 2.50)
Palau	361.01 (221.97, 533.21)	110.02 (73.89, 152.20)	3.98 (5.91, 2.43)	1.21 (1.68, 0.81)	3.89 (6.15, 2.23)	1.52 (2.38, 0.88)
Palestine	510.63 (318.83, 777.31)	48.41 (30.40, 72.18)	5.64 (8.60, 3.52)	0.53 (0.80, 0.33)	4.79 (7.36, 2.92)	0.88 (1.33, 0.54)
Panama	547.14 (431.20, 675.57)	154.04 (104.42, 209.59)	6.05 (7.48, 4.77)	1.70 (2.31, 1.15)	4.92 (8.03, 2.47)	2.12 (3.51, 1.08)
Papua New Guinea	1655.22 (1110.98, 2308.56)	665.81 (436.36, 971.55)	18.36 (25.66, 12.32)	7.36 (10.79, 4.82)	8.40 (12.73, 5.35)	5.82 (8.93, 3.64)
Paraguay	920.62 (671.38, 1208.88)	105.74 (59.81, 167.00)	10.04 (13.25, 7.31)	1.17 (1.85, 0.66)	20.02 (30.69, 11.80)	0.61 (0.97, 0.32)
Peru	1542.82 (1051.99, 2085.83)	63.16 (31.68, 104.70)	17.04 (23.03, 11.61)	0.70 (1.16, 0.35)	14.87 (22.90, 8.60)	0.61 (0.99, 0.35)
Philippines	1116.42 (799.19, 1428.26)	234.12 (166.07, 312.27)	12.27 (15.70, 8.78)	2.56 (3.43, 1.81)	16.80 (26.04, 9.84)	4.23 (6.98, 2.38)
Poland	74.99 (59.43, 91.38)	26.45 (19.87, 33.82)	0.81 (0.99, 0.64)	0.29 (0.37, 0.22)	2.23 (3.69, 1.27)	0.38 (0.58, 0.23)
Portugal	85.80 (69.37, 102.84)	11.10 (8.36, 14.06)	0.89 (1.08, 0.72)	0.09 (0.11, 0.07)	5.90 (9.25, 3.33)	3.15 (5.02, 1.84)
Puerto Rico	88.47 (65.13, 108.65)	35.45 (26.50, 45.89)	0.97 (1.20, 0.71)	0.38 (0.50, 0.28)	1.39 (2.25, 0.75)	1.42 (2.15, 0.84)
Qatar	159.29 (105.75, 225.81)	21.09 (14.70, 28.94)	1.73 (2.47, 1.14)	0.21 (0.30, 0.15)	3.86 (5.96, 2.20)	1.92 (3.03, 1.17)

Republic of Korea	85.69 (60.34, 119.44)	9.70 (6.54, 13.85)	0.94 (1.32, 0.66)	0.10 (0.15, 0.07)	1.11 (1.78, 0.67)	0.64 (1.07, 0.35)
Republic of Moldova	518.27 (391.92, 655.85)	92.92 (64.72, 129.43)	5.63 (7.16, 4.22)	0.99 (1.39, 0.67)	12.92 (19.06, 8.51)	4.18 (6.22, 2.75)
Romania	758.45 (555.09, 983.26)	105.76 (75.32, 136.71)	8.41 (10.90, 6.15)	1.17 (1.51, 0.83)	3.41 (4.91, 2.15)	0.99 (1.50, 0.65)
Russian Federation	243.55 (199.44, 284.09)	35.55 (28.92, 42.05)	2.58 (3.02, 2.10)	0.38 (0.45, 0.31)	12.13 (18.91, 7.13)	1.87 (2.94, 1.12)
Rwanda	1426.84 (901.09, 2033.66)	142.56 (83.78, 220.55)	15.82 (22.60, 9.95)	1.58 (2.44, 0.92)	8.09 (12.46, 4.79)	1.01 (1.54, 0.61)
Saint Kitts and Nevis	590.21 (476.69, 706.56)	135.59 (93.60, 184.15)	6.54 (7.84, 5.29)	1.50 (2.04, 1.03)	3.85 (5.74, 2.37)	1.45 (2.18, 0.89)
Saint Lucia	347.41 (264.56, 434.57)	74.24 (47.94, 109.56)	3.83 (4.81, 2.92)	0.81 (1.20, 0.52)	3.48 (5.20, 2.11)	1.30 (1.97, 0.82)
Saint Vincent and the Grenadines	582.19 (423.01, 725.43)	85.73 (58.21, 120.76)	6.44 (8.05, 4.68)	0.94 (1.33, 0.64)	4.92 (7.40, 3.10)	1.02 (1.53, 0.66)
Samoa	237.09 (141.90, 356.14)	71.27 (42.67, 114.36)	2.60 (3.93, 1.54)	0.78 (1.26, 0.46)	3.61 (5.69, 2.17)	1.68 (2.64, 1.01)
San Marino	16.33 (10.72, 24.16)	4.26 (2.48, 6.52)	0.14 (0.23, 0.09)	0.02 (0.03, 0.01)	3.36 (5.77, 1.66)	2.89 (4.92, 1.44)
Sao Tome and Principe	2023.61 (1423.65, 2763.86)	112.06 (67.70, 178.28)	22.45 (30.71, 15.77)	1.24 (1.98, 0.75)	9.79 (14.70, 5.93)	0.55 (0.84, 0.35)
Saudi Arabia	446.69 (290.18, 687.08)	21.47 (13.56, 32.86)	4.88 (7.55, 3.14)	0.23 (0.36, 0.14)	8.72 (13.35, 5.18)	0.87 (1.38, 0.53)
Senegal	3343.60 (2398.01, 4400.33)	359.69 (209.00, 540.60)	37.12 (48.85, 26.60)	3.98 (6.00, 2.30)	14.83 (21.67, 9.18)	2.56 (3.95, 1.60)
Serbia	159.79 (106.21, 218.69)	21.22 (14.34, 29.50)	1.76 (2.42, 1.17)	0.23 (0.32, 0.15)	1.52 (2.32, 0.92)	0.96 (1.53, 0.57)
Seychelles	207.67 (143.95, 280.16)	115.84 (76.37, 165.88)	2.27 (3.08, 1.57)	1.27 (1.83, 0.83)	3.62 (5.51, 2.27)	1.76 (2.56, 1.10)
Sierra Leone	5221.65 (3269.96, 7781.19)	586.00 (345.38, 871.37)	58.00 (86.56, 36.28)	6.51 (9.69, 3.83)	17.19 (24.88, 11.22)	2.46 (3.72, 1.54)
Singapore	125.29 (98.40, 152.24)	11.89 (8.97, 15.33)	1.38 (1.68, 1.08)	0.13 (0.16, 0.10)	1.22 (1.84, 0.76)	0.40 (0.64, 0.23)
Slovakia	155.32 (109.99, 206.33)	39.64 (26.63, 54.19)	1.72 (2.29, 1.22)	0.44 (0.60, 0.29)	0.62 (0.99, 0.37)	0.45 (0.72, 0.27)
Slovenia	75.41 (53.68, 97.75)	21.15 (15.08, 28.10)	0.83 (1.08, 0.59)	0.22 (0.30, 0.16)	1.09 (1.76, 0.63)	1.29 (2.02, 0.76)
Solomon Islands	667.19 (430.15, 995.26)	159.91 (101.29, 236.34)	7.38 (11.02, 4.74)	1.77 (2.61, 1.11)	5.11 (7.98, 3.13)	1.51 (2.33, 0.95)
Somalia	6429.69 (4108.57, 8988.93)	2169.65 (1354.03, 3019.11)	71.48 (100.01, 45.61)	24.12 (33.57, 15.04)	16.51 (24.75, 10.26)	5.06 (7.37, 3.12)
South Africa	3548.47 (2836.47, 4150.43)	911.74 (687.48, 1199.87)	39.37 (46.07, 31.47)	10.11 (13.31, 7.60)	17.76 (26.54, 10.79)	5.16 (7.96, 3.14)
South Sudan	6038.63 (3818.06, 8696.93)	2204.11 (1387.42, 3279.45)	67.12 (96.75, 42.41)	24.48 (36.47, 15.40)	13.71 (19.87, 8.78)	7.20 (10.32, 4.64)
Spain	28.47 (22.47, 33.99)	10.72 (8.05, 13.65)	0.27 (0.33, 0.22)	0.08 (0.10, 0.06)	4.12 (6.66, 2.35)	3.72 (6.06, 2.12)
Sri Lanka	244.22 (170.59, 328.36)	16.84 (10.56, 25.32)	2.65 (3.58, 1.85)	0.18 (0.27, 0.11)	6.49 (9.82, 4.04)	0.96 (1.52, 0.60)
Sudan	3337.93 (1966.03, 5145.26)	150.07 (74.24, 355.52)	37.01 (57.11, 21.76)	1.62 (3.91, 0.78)	17.48 (25.48, 11.06)	4.50 (6.81, 2.74)
Suriname	1549.24 (1097.02, 1991.22)	322.16 (194.90, 484.08)	17.18 (22.11, 12.14)	3.57 (5.37, 2.15)	7.91 (11.82, 5.00)	2.03 (3.09, 1.30)
Sweden	12.15 (9.14, 15.17)	6.30 (4.64, 8.19)	0.11 (0.14, 0.08)	0.04 (0.06, 0.03)	2.29 (4.04, 1.16)	2.31 (3.94, 1.25)
Switzerland	35.38 (28.05, 43.73)	15.57 (11.02, 21.03)	0.29 (0.35, 0.22)	0.07 (0.09, 0.05)	9.67 (15.08, 5.68)	9.11 (14.29, 5.46)
Syrian Arab Republic	452.40 (292.66, 689.90)	22.53 (13.05, 33.39)	4.92 (7.57, 3.17)	0.23 (0.35, 0.13)	11.07 (16.79, 6.99)	1.54 (2.33, 0.96)

Taiwan (Province of China)	68.01 (50.40, 85.96)	14.78 (10.37, 19.67)	0.71 (0.91, 0.52)	0.12 (0.16, 0.08)	4.46 (6.89, 2.66)	4.26 (6.77, 2.51)
Tajikistan	2573.97 (1771.60, 3478.25)	741.58 (477.54, 1028.99)	28.52 (38.59, 19.59)	8.23 (11.44, 5.28)	15.81 (23.12, 9.69)	3.40 (5.00, 2.10)
Thailand	608.80 (340.30, 934.53)	120.35 (87.23, 153.06)	6.62 (10.24, 3.60)	1.29 (1.66, 0.93)	14.33 (21.76, 8.80)	4.21 (6.35, 2.51)
Timor-Leste	4424.86 (2823.74, 6051.57)	458.99 (301.10, 634.68)	49.14 (67.24, 31.30)	5.06 (7.01, 3.31)	16.39 (24.05, 10.32)	5.31 (8.25, 3.14)
Togo	3086.88 (2094.28, 4376.30)	498.11 (291.18, 790.51)	34.25 (48.58, 23.19)	5.51 (8.77, 3.20)	13.83 (20.13, 9.09)	3.53 (5.23, 2.23)
Tokelau	339.58 (218.19, 505.66)	583.64 (375.21, 831.77)	3.76 (5.62, 2.41)	6.50 (9.27, 4.18)	2.79 (4.41, 1.67)	0.94 (1.55, 0.55)
Tonga	217.31 (144.50, 310.53)	66.13 (39.32, 100.64)	2.39 (3.41, 1.58)	0.73 (1.11, 0.43)	3.16 (4.79, 1.90)	0.93 (1.46, 0.56)
Trinidad and Tobago	312.60 (239.11, 394.06)	73.65 (48.69, 104.28)	3.46 (4.37, 2.64)	0.81 (1.15, 0.53)	2.43 (3.69, 1.48)	0.85 (1.28, 0.53)
Tunisia	640.93 (394.49, 939.77)	61.11 (35.01, 91.71)	7.03 (10.35, 4.27)	0.66 (1.00, 0.37)	10.16 (15.72, 6.10)	1.92 (2.88, 1.15)
Turkey	1234.70 (760.80, 1863.11)	46.22 (29.31, 66.58)	13.62 (20.65, 8.32)	0.50 (0.73, 0.32)	12.65 (19.82, 7.54)	1.33 (2.06, 0.80)
Turkmenistan	2277.95 (1557.12, 2944.20)	212.24 (119.31, 334.52)	25.25 (32.69, 17.25)	2.35 (3.71, 1.32)	13.13 (19.17, 8.23)	1.06 (1.55, 0.66)
Tuvalu	1271.67 (789.40, 1878.08)	138.68 (84.48, 201.83)	14.12 (20.88, 8.75)	1.53 (2.24, 0.93)	5.45 (8.52, 3.19)	1.19 (1.81, 0.73)
Uganda	2358.63 (1203.02, 3613.54)	228.09 (120.11, 396.77)	26.15 (40.14, 13.30)	2.52 (4.40, 1.32)	14.00 (20.92, 8.54)	2.45 (3.68, 1.55)
Ukraine	158.07 (115.43, 198.44)	28.80 (20.36, 38.23)	1.66 (2.11, 1.19)	0.31 (0.41, 0.22)	9.26 (14.57, 5.47)	1.03 (1.63, 0.61)
United Arab Emirates	172.58 (122.76, 240.50)	26.76 (17.88, 37.06)	1.88 (2.63, 1.32)	0.29 (0.40, 0.19)	3.70 (5.85, 2.19)	1.17 (1.90, 0.64)
United Kingdom	33.66 (27.08, 39.95)	10.78 (8.35, 13.36)	0.34 (0.41, 0.27)	0.10 (0.13, 0.08)	3.10 (5.41, 1.66)	1.67 (2.77, 0.96)
United Republic of Tanzania	4071.39 (2727.79, 5475.81)	380.64 (238.03, 571.16)	45.19 (60.88, 30.25)	4.23 (6.35, 2.64)	15.48 (22.62, 10.11)	1.60 (2.42, 1.02)
United States of America	43.75 (36.69, 50.38)	10.14 (8.19, 12.16)	0.45 (0.52, 0.38)	0.11 (0.13, 0.09)	3.09 (5.06, 1.69)	0.36 (0.55, 0.22)
United States Virgin Islands	121.05 (81.07, 165.31)	21.93 (13.43, 32.55)	1.33 (1.82, 0.89)	0.24 (0.36, 0.14)	1.67 (2.55, 1.00)	0.53 (0.88, 0.31)
Uruguay	275.74 (220.91, 331.60)	38.25 (27.50, 51.02)	2.99 (3.62, 2.39)	0.42 (0.56, 0.30)	7.80 (11.59, 4.77)	0.74 (1.13, 0.44)
Uzbekistan	1617.52 (1131.68, 2101.27)	289.63 (159.11, 431.95)	17.90 (23.27, 12.51)	3.22 (4.80, 1.77)	10.30 (15.10, 6.58)	0.42 (0.62, 0.26)
Vanuatu	683.53 (430.13, 1008.25)	197.89 (121.64, 294.14)	7.54 (11.15, 4.73)	2.18 (3.26, 1.32)	7.33 (11.46, 4.44)	2.74 (4.14, 1.74)
Venezuela (Bolivarian Republic of)	1255.41 (1053.24, 1452.38)	185.18 (114.93, 259.84)	13.94 (16.14, 11.69)	2.05 (2.88, 1.27)	5.31 (8.26, 3.01)	1.44 (2.30, 0.75)
Viet Nam	683.74 (422.37, 953.90)	92.39 (49.80, 145.08)	7.48 (10.49, 4.58)	1.00 (1.59, 0.53)	12.10 (18.18, 7.53)	2.68 (4.14, 1.60)
Yemen	4784.85 (3145.99, 6568.72)	295.27 (166.53, 482.00)	53.11 (73.00, 34.91)	3.22 (5.30, 1.79)	19.24 (28.44, 12.03)	6.22 (9.55, 3.94)
Zambia	4311.02 (2878.19, 5807.50)	290.08 (172.09, 433.28)	47.88 (64.57, 31.92)	3.20 (4.79, 1.90)	17.23 (25.58, 11.16)	2.81 (4.18, 1.79)
Zimbabwe	1533.56 (1100.35, 1920.75)	490.98 (309.30, 704.21)	16.97 (21.27, 12.15)	8.87 (12.02, 5.84)	10.55 (15.96, 6.76)	2.74 (4.10, 1.78)
